# Sudden Sensorineural Hearing Loss in the COVID-19 Pandemic: A Systematic Review and Meta-Analysis

**DOI:** 10.3390/diagnostics12123139

**Published:** 2022-12-12

**Authors:** Andrea Frosolini, Leonardo Franz, Antonio Daloiso, Cosimo de Filippis, Gino Marioni

**Affiliations:** 1 Audiology Unit, Department of Neuroscience DNS, University of Padova, 31100 Treviso, Italy; 2 Maxillofacial Surgery Unit, Department of Medical Biotechnologies, University of Siena, 53100 Siena, Italy; 3 Otolaryngology Section, Department of Neuroscience DNS, University of Padova, 35100 Padova, Italy; 4 Guided Therapeutics (GTx) International Scholarship Program, Techna Institute, University Health Network (UHN), Toronto, ON M5G2C4, Canada

**Keywords:** sudden sensorineural hearing loss, SSNHL, SARS-CoV-2, COVID-19

## Abstract

Introduction: Accumulating data indicate that patients with COVID-19 could be affected by sudden sensorineural hearing loss (SSNHL). The aim of the study was to analyze the epidemiological and clinical trend of SSNHL occurrence during the COVID-19 pandemic by applying a systematic review and meta-analysis approach. Methods: PubMed, Scopus, Web of Science, ScienceDirect, and Cochrane databases were searched. Results: The seven included studies had adequate relevance to the topic and the quality was fair. The mean age at SSNHL onset ranged from 39.23 to 62.18 years during the pandemic year period (PYP); a meta-analysis of four studies comparing these data with those of previous periods in the same institutions found a younger age during the PYP (pooled mean −0.2848). The heterogeneity was high (76.1935%) and no frank asymmetry was observed in the funnel plot. The SARS-CoV-2 positivity rate of SSNHL patients ranged from 0% to 57.53%. Standard steroid treatments were applied without significant adverse effects. Comprehensively, hearing improvement was achieved for more than half of the cases. No studies reported long-term follow-up data. Conclusions: Further prospective analyses on large series and a long-term follow up on COVID-related SSNHL cases are necessary to address the open questions regarding the causative link between COVID-19 infection and SSNHL.

## 1. Introduction

Sudden sensorineural hearing loss (SSNHL) is a hearing loss of at least 30 dB at three contiguous frequencies occurring within 72 h [1]. SSNHL is one of the most frequent pathologies seen in the ENT and Audiology units, and in the United States in 2006–2007, its annual incidence was 27 cases per 100,000, with about 66,000 new cases per year [2]. SSNHL could be due to various causes: vascular events, viral infection, or autoimmune disorders. In most cases, SSNHL is classified as idiopathic. However, nowadays, virus infections should be screened more frequently in order to better understand the disease and establish more suitable treatment to restore patients’ hearing [3].

SARS-CoV-2 was primarily identified in December 2019. The World Health Organization (WHO) declared the global outbreak of coronavirus in March 2020 and coined the term “Coronavirus Disease 2019 (COVID-19)”. Globally, as of 29 July 2022, there have been 572,239,451 confirmed cases of COVID-19 and more than 6 million deaths [4]. Typical symptoms of COVID-19 comprise fever, cough, fatigue, shortness of breath, and smell and taste disorders [5]. Furthermore, inner ear symptoms, tinnitus, and balance disorders were initially mentioned in sporadic case reports [6,7,8,9]. Since its early descriptions [3,10], SSNHL in COVID-19 patients has been regarded as the possible result of virus-induced damage to cochlear hair cell function. This was also supported by the identification of the virus presence in the middle ear and mastoid [11], possibly leading to an invasion of the labyrinth compartment [10].

The currently accumulating data indicate that patients with COVID-19 could be affected by SSNHL [3,12,13,14]. Nonetheless, the absolute number of patients presenting SSNHL during the pandemic period seems to have decreased, while the percentage of patients diagnosed compared to the total number of those evaluated before and during the pandemic remain approximately unchanged [15,16] or even augmented [17], due to a reduction in outpatient visits. Several case reports and original studies have been published on this subject to date, whereas well-structured systematic reviews with meta-analysis are lacking.

The main aim of this study was to analyze the epidemiological and clinical trend of SSNHL during the COVID-19 pandemic by applying a systematic literature review and a meta-analysis approach. A secondary aim was to evaluate possible differences in the epidemiological landscape between pandemic and pre-pandemic periods.

## 2. Materials and Methods

### 2.1. Protocol Registration

The systematic review and meta-analysis protocol of the present investigation was registered on PROSPERO, an international prospective register of systematic reviews (Center for Reviews and Dissemination, University of York, York, UK), in July 2022 (registry number CRD42022349286).

### 2.2. Electronic Database Search

A search of the English literature published on the databases PubMed, Scopus, Web of Science, ScienceDirect, and Cochrane was performed according to the Preferred Reporting Items for Systematic Reviews and Meta-Analyses (PRISMA) recommendations [18]. The final search was conducted on 31 July 2022. The following keywords were used: “Sudden Sensorineural Hearing Loss”; “SSNHL”; “SARS-CoV-2”; “COVID-19”. The keywords were combined accordingly on the aforementioned databases. The reference lists of all of the included articles were accurately screened in order to identify other pertinent studies. The “Related articles” options present on the PubMed and Scopus homepages were also considered. The references were exported to Zotero bibliography manager (v6.0.10, Center for History and New Media, George Mason University, Fairfax, VA, USA) to remove duplicates and they were then transposed into an Excel (Microsoft Excel 2019 for Windows 10) spreadsheet for eligibility screening.

### 2.3. Inclusion and Exclusion Criteria

Investigations were included only if the following general criteria were met: (i) original research conducted at Audiology/Otolaryngology referral centers; (ii) SSNHL cases enrolled during the pandemic period; (iii) detailed information on the epidemiology of reported cases, including characteristics of the swabbing procedure for SARS-CoV-2 infection, when performed. The exclusion criteria were: (i) articles in the form of a case report, editorial, survey, letter to the editor, or review; (ii) animal model studies, and (iii) non-English language used.

### 2.4. Data Extraction and Quality Assessment

The authors analyzed the data from the available literature. The included studies were analyzed to extract the available data and ensure eligibility for all patients. The risk of bias was considered for all included studies. Any disagreements about the inclusion/exclusion of investigations were resolved by a discussion among the study team members. The quality rating of each study was categorized as poor, fair, or good, according to the National Institutes of Health Quality Assessment Tool for Observational Cohorts and Cross-Sectional Studies [19].

### 2.5. Statistical Analysis

The analysis was performed using the standardized mean difference as the outcome measure and a random-effects model was fitted to the data. The amount of heterogeneity was estimated using the restricted maximum likelihood estimator, the Q-test for heterogeneity, and the I² statistic [20].

A prediction interval for the true outcomes was also provided. Studentized residuals and Cook’s distances were used to examine whether studies may be outliers and/or influential in the context of the model. The rank correlation test and regression test, using the standard error of the observed outcomes as a predictor, were used to check for funnel plot asymmetry.

The Jamovi (version 2.3) computer software (The Jamovi Project 2022, Sidney, Australia) for macOS Big Sur was used [21,22].

## 3. Results

### 3.1. Retrieving Studies

**Figure 1:**
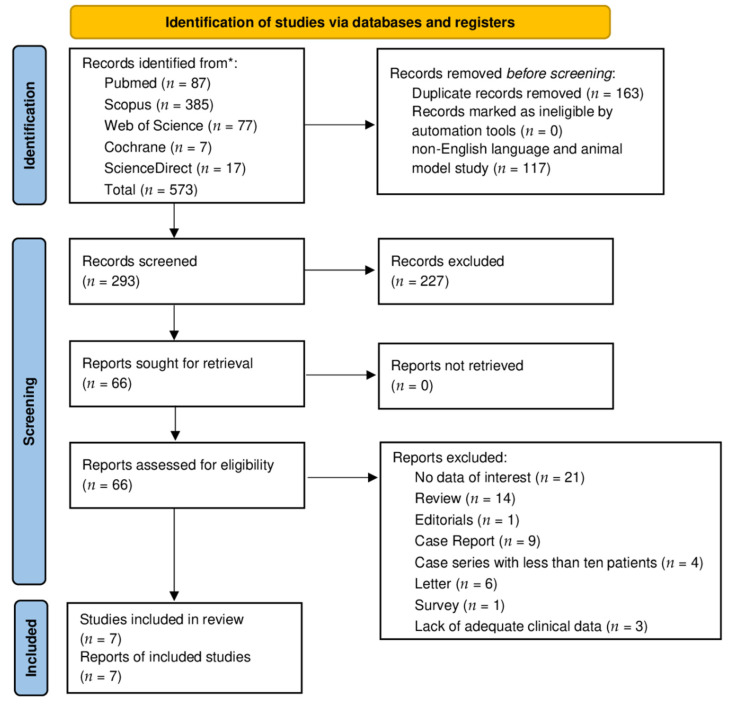
PRISMA [18] diagram resembling the electronic database search and inclusion/exclusion process of the review. Legend: * date of last search: 31 July 2022.

A total of 573 titles were retrieved from the database search and from cross-reference checking (87 from PubMed; 385 from Scopus; 77 from Web of Science; 17 from ScienceDirect, and 7 from Cochrane). After the removal of duplicates, non-English language papers, and animal model studies, 293 manuscripts were identified. Selection based on title and abstract screening led to the exclusion of 227 studies. The 66 remaining studies potentially relevant to the topic were accurately examined and, after full-text screening and the application of the inclusion/exclusion criteria, seven articles were included in the qualitative synthesis [17,23,24,25,26,27,28]. The PRISMA chart (Figure 1) summarizes the article inclusion process in this systematic review. Subsequently, four articles were included in the quantitative synthesis [17,23,26,27].

### 3.2. Quality Assessment

All included studies had adequate relevance to the subject of this systematic review. None were a randomized controlled trial; five studies were retrospective [17,23,24,25,26], whereas two were prospective [27,28].

According to the National Institutes of Health Quality Assessment Tool for Observational Cohorts and Cross-Sectional Studies [19], only one study was rated as good [17], five studies as fair [23,24,25,26,27], and one as poor [28]. The characteristics of the included studies are summarized in Table 1.

### 3.3. Qualitative Synthesis

Study population, diagnostic workout, treatment administered, and outcome were considered during data extraction for each included study. As shown in Table 1, all research groups included the SSNHL cases during the pandemic year period (PYP). Moreover, four out of ten studies also considered the cases that occurred during the pre-pandemic year period (pre-PYP) as controls [17,23,26,27].

Epidemiological data of SSNHL during PYP vs. pre-PYP were reported by two research groups, with different methods: number of SSNHL cases over total outpatient visits [17] or incidence ratio of SSNHL cases per 100,000 residents [23]. In both cases, the reported values were higher during the PYP [17,23] (see Table 1).

The characteristics of SSNHL patients in the PYP are reported in Table 2, while the characteristics of SSNHL cases in pre-PYP (control groups) are shown in Table 3. In the PYP, the described SSNHL cases regarded patients with mean ages ranging from 39.23 [25] to 62.18 [24] years, with an overall male prevalence (181 males and 154 females). In the pre-PYP, the mean age of the SSNHL patients ranged from 50.73 [27] to 67.2 [23] years; the gender distribution also showed a male prevalence (156 males and 113 females).

The clinical presentation, apart from sudden hearing loss, mainly consisted of associated tinnitus and dizziness. These data were reported by four investigations [17,24,25,28] considering SSNHL in the PYP and one [17] also analyzing the pre-PYP.

A SARS-CoV-2 test with diagnostic purposes was performed at the time of SSNHL diagnosis in three studies [17,23,27]. No positive swabs were found in one series [27], whereas the maximum positivity percentage was 57.35% [23]. Globally, 28.05% of the swabs performed were positive (41 out of 115 cases). In the other two investigations, the study groups comprised only COVID-19-positive patients [25,28].

Regarding pure tone audiometry, in the PYP, the pre-treatment PTA in the SSNHL cases was evaluated by the investigations [17,24,25], and ranged from 50.91 ± 11.77 dB [25] to 68.77 ± 23.32 dB [24]. Only two studies reported the post-treatment PTA: 40.24 ± 15.69 dB in the Yaseen et al. [25] investigation and 50.4 ± 25.6dB in Parrino et al. [17]. In the pre-PYP control group, only Parrino et al. [17] evaluated pre- and post-treatment PTA.

Contrast-enhanced MRI was performed on SSNHL patients enrolled by two research groups during the PYP [25,28] to evaluate the state of the auditory pathways. Swain et al. [28] reported an enhancement in the affected cochlea in 10 out of 16 patients, thus highlighting a possible risk of ossification; Yaseen et al. [25] found no abnormalities. In the control groups of SSNHL that occurred during the pre-PYP, none of the authors reported alterations at MRI.

Considering SSNHL patients in the PYP, five out of seven studies described different therapeutic protocols, as summarized in Table 2. The single-strategy treatment consisted of a course of oral (OS) [23,28], intravenous (IVS) [17], or intratympanic steroid administration (ITS) [24]. A combination therapy of ITS plus OS was proposed for subgroups of patients by two investigators [17,25]. Tsuda et al. [24] applied hyperbaric oxygen salvage therapy (HOT) for patients who had not recovered after OS and ITS. Considering pre-PYP control groups, only one study reported administered therapies [17] (see Table 3). The outcome was reported by five studies: no definition of the criteria was given by three studies [25,27,28], and only one relied on Siegel’s criteria [17]. Tsuda et al. defined complete recovery as “hearing threshold at all frequencies recovered within 20 dB”, marked recovery as “hearing level improvement ≥30 dB”, slight recovery as “hearing level improvement ≥10 dB but <30 dB”, no remarkable change as “hearing level improvement <10 dB” [24]. The best treatment outcomes were reported by Yaseen et al. (84% of the patients recovered) [25], while the worst outcomes were reported by Aslan et al. (52.38% of the patients recovered) [27] in the PYP. In the pre-PYP control groups, outcomes were reported by Parrino et al. (70.37% of the patients recovered) [17] and Aslan et al. (51.04% of the patients recovered) [27]. No authors reported an audiological long-term follow-up for the enrolled patients.

### 3.4. Quantitative Analysis

**Figure 2:**
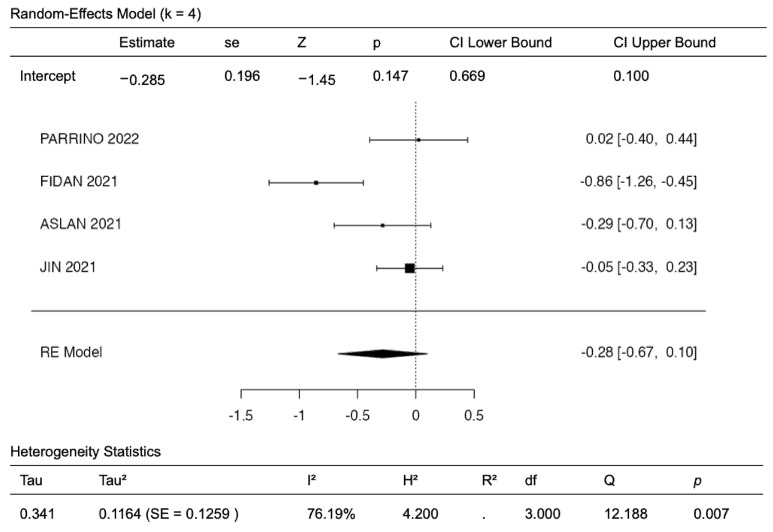
Forest plot showing mean differences in age of onset of SSNHL patients at single institutions in the pandemic year period vs. pre-pandemic year period.

A total of four studies [17,23,26,27] were included in the analysis of age at onset of SSNHL during PYP vs. pre-PYP (Figure 2). The age at SSNHL onset during PYP seemed tendentially lower without reaching statistical significance, as the observed standardized mean differences ranged from −0.8559 to 0.0242, with the majority of the estimates being negative (75%) [23,26,27]. The estimated average standardized mean difference, based on the random-effects model, was −0.2848 (95% CI: −0.6695 to 0.0999; z = −1.4508, *p* = 0.1468). The heterogeneity was high (I² = 76.1935%). A 95% prediction interval for the true outcomes is given by −1.0561 to 0.4866.

**Figure 3:**
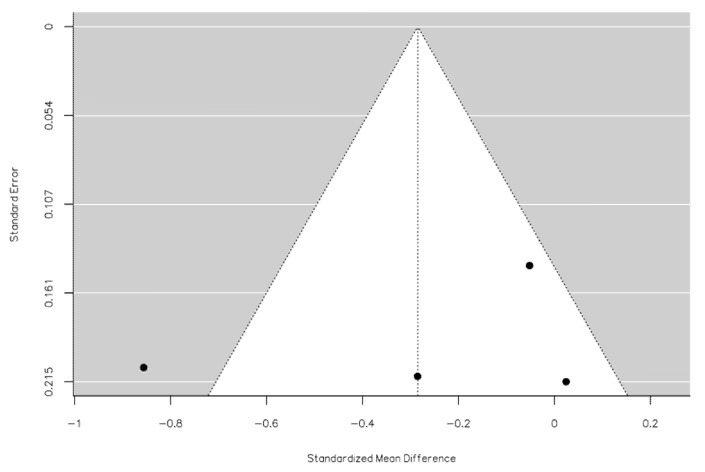
Funnel plot of meta-analysis regarding age of onset of SSNHL patients in the pandemic year period vs. pre-pandemic year period. Black dots identify each study’s characteristics in terms of standard error and standardized mean difference.

An examination of the studentized residuals revealed that one study [23] had a value higher than ±2.4977 and may be a potential outlier in the context of this model (Figure 3). According to Cook’s distances, none of the studies could be considered to be overly influential. Neither the rank correlation nor the regression test indicated any funnel plot asymmetry (*p* = 1.0000 and *p* = 0.6271, respectively; Figure 3).

## 4. Discussion

Since its outbreak, the COVID-19 pandemic has led to a major healthcare and economic burden. Most symptomatic patients experience mild respiratory illness, but the long-term complications of this infection still need to be characterized.

### 4.1. Audio-Vestibular Symptoms in COVID-19: Hypotheses on Patho-Physiology

The link between sensorineural hearing loss and infection with viruses such as mumps, cytomegalovirus (CMV), and Epstein–Barr virus has been established [29,30,31], particularly in the pediatric population. For instance, SSNHL is thought to be a consequence of an immune-mediated response triggered by CMV [30]. On the other hand, studies highlighted how rubella directly damaged the cochlear epithelium and stria vascularis [32], and varicella zoster virus impaired the vestibulocochlear nerve [33]. Moreover, sporadic cases of hearing loss have been reported due to viruses with neuro-invasive potential, such as West Nile virus [34].

COVID-19’s otorhinolaryngological symptoms include sore throat, rhinorrhea, nasal congestion, throat congestion, tonsil edema, enlarged cervical lymph nodes, and anosmia, which, in most cases, resolve without sequelae [35]. Specifically, in the otologic field, the available evidence suggests that COVID-19 could cause a spectrum of audiovestibular symptoms, including various degrees of sensorineural hearing loss, equilibrium disorders, and tinnitus [36,37].

Previous systematic reviews have described possible neuro-invasive actions causing hearing loss by SARS-CoV-2 infection [38,39,40]. It has been suggested that SARS-CoV-2 binds to the ACE-2 receptor, which was recently seen to be expressed in the epithelial cells of the middle ear, as well as in the stria vascularis and spiral ganglion in mice [41]. Moreover, SARS-CoV-2 infection causes an inflammatory response and an increase in cytokines known to be potentially harmful to cochlear structures [7,42,43]. On the other hand, the use of ototoxic medications, such as chloroquine, in some COVID-19 patients may have acted as a confounding factor, making an accurate differentiation between ototoxicity and an actual virus-related SSNHL hard to achieve [44]. However, the consistency of the hypotheses around the causative link between COVID-19 and audiovestibular symptoms may be affected by possible biases resulting from the symptom reporting methods employed in the published studies, as suggested by Saunders et al. [45]. In fact, the nocebo effect might have led some COVID-19 patients to complain, for the first time during the infection period, about symptoms they had experienced before.

The paucity of available clinical data was a main result from our systematic review, as we will discuss later, in accordance with what was retrieved by other researchers during the COVID-19 pandemic [46]. These limitations affected the approach to the comparison of symptoms incidence in the pandemic and pre-pandemic period to address the question of a possible direct link between COVID-19 and audiovestibular disorders.

### 4.2. Incidence of Audiological Symptoms during the Pandemic Period

According to a previous experience by our clinical research group [17], an increase was found in the absolute number of cases with acute audiovestibular disorders during the COVID-19 pandemic period compared to previous ones, without reaching statistical significance. Similar results were found by a retrospective observational Turkish study, which described a higher incidence of SSNHL during the 2020 COVID-19 pandemic wave, compared to the same period of the previous non-pandemic year [23]. Furthermore, in order to evaluate the impact of COVID-19 on otolaryngology diseases, a large tertiary hospital study in China found an increase in SSNHL incidence during the COVID-19 pandemic [26]. The Aslan et al. investigation [27] found no difference in SSNHL diagnosed in the periods from January 2019 to January 2020 (42 cases) and April 2020 to April 2021 (49 cases). Based on the studies included in the present review, the evidence about incidence changes in SSNHL between the pre-pandemic and pandemic periods were discordant and not conclusive (see also Table 1). This heterogeneity also reflects the overall low quality of the available studies, which reported limited clinical and audiological data (see also Table 1 and Table 2) and were mostly based on non-homogeneous retrospective series.

Several studies potentially relevant to this topic were excluded from the present investigation due to a lack of adequate clinical data. Among the original studies excluded from our review, Chao et al. [16] analyzed the epidemiology trend of audiovestibular disorder diagnoses at their institute: the incidence of SSNHL from 2016 (2.6%) to 2020 (2.1%) showed no statistically significant difference. Chari et al. [15] experienced a decrease in the absolute number of SSNHL diagnoses during the COVID-19 outbreak (19 cases) compared to the pre-pandemic period (71 cases). However, the proportion of SSNHL diagnoses over total audiological evaluations during the pandemic period (1.91%) was higher than the pre-pandemic period (1.77%) [15].

### 4.3. Demographics and Clinical Features of SSNHL Cases during the Pandemic Period

The evaluation of age at SSNHL onset in the PYP compared to the pre-PYP was an object of the quantitative meta-analysis in this investigation. The pooled analysis revealed a trend towards a slightly younger age in SSNHL patients during the PYP, although this result should be carefully regarded, in consideration of the high heterogeneity of the included studies. However, no frank asymmetry in the funnel plot, potentially standing for publication bias, was revealed. Considering the studies included in the meta-analysis, a younger age at onset of SSNHL during the PYP was found by three investigations [23,26,27], while the remainder [17] did not show any substantial age difference.

A male prevalence was observed in all considered studies [17,23,24,25,26,27,28], in accordance with what is already known for SSNHL [1].

Regarding hearing loss severity, an Italian study [17] found higher PTA values at onset in the PYP, along with a higher rate of associated vestibular symptoms, compared to the pre-PYP.

### 4.4. Clinical Features of SSNHL in COVID-19 Patients

In addition to the overall features of SSNHL during the pandemic period, some considerations may be drawn regarding cochlear symptoms in the specific population of COVID-19 patients.

The SARS-CoV-2 positivity rate of SSNHL patients ranged from 0% [27] to 57.53% [23], being 29.05% overall. Such variability could probably be due to the between-study heterogeneity in terms of geographic area, demographics, and observation period, considering the differences in SARS-CoV-2 incidence in the general population according to seasonality and periodic pandemic waves.

Regarding hearing loss onset, an Iraq study based on a series of 30 COVID-19 patients who also developed SSNHL showed onset within 9 days from initial COVID-19 diagnosis in all cases (mean 4.1 ± 2.9 days) [25]. The same study described bilateral hearing loss and tinnitus as the most commonly associated symptoms [25].

### 4.5. SSNHL Treatment, Outcome, and Follow-up in the Pandemic Scenario

The present systematic review also focused on the treatment modalities and outcomes of SSNHL during the pandemic period. The preferred treatments were OS, ITS, IVS, and HOT, as singular or combination therapies, according to various protocols. No significant adverse effects have been reported. Tsuda et al. [24] compared the efficacy of intratympanic vs. intravenous steroid therapy as the initial treatment for SSNHL during the COVID-19 pandemic in 68 patients, finding no significant outcome differences. Based on these equivalent results, Tsuda et al. [24] proposed ITS as a reasonable first-line treatment to avoid the possible side effects of a systemic steroid treatment.

Outcomes were reported with standardized Siegel’s criteria by only one research group [17] and three out of five studies did not report any evaluation criteria [25,27,28]. Overall, considering all of the treated patients, an audiological improvement was achieved for more than half of the subjects [17,24,25,27,28].

Given the relatively recent spread of COVID-19, none of the considered studies could report any audiological data based on long-term follow-up. Further prospective studies are mandatory to address this topic.

### 4.6. Quality of Evidence on SSNHL during COVID-19 Pandemic

The only previous systematic reviews available in the literature on this topic were two analyses of case reports [47,48] published in 2021, at earlier stages of clinical research on COVID-19, and one systematic review recently published that settled general inclusion/exclusion criteria (mainly related to the type of manuscript without considering any diagnostic standards) and only applied a qualitative approach [49].

The present investigation is the first systematic review and meta-analysis regarding SSNHL during COVID-19 developed using a well-defined protocol and exclusion/inclusion criteria. The main limitation is related to the overall low quality of the available literature (see also Table 1), which in most cases did not provide enough audiological and radiological data, making it difficult to achieve conclusive results. In light of the possibility of future viral pandemics, the need to develop better data ecosystems has been stressed, to obtain higher quality research in an emergency context as well [50]. With reference to a specific clinical manifestation of viral infections, such as SSNHL, the development of epidemic-specific data collection systems could give the opportunity to obtain stronger clinical evidence, potentially shedding light on causative relationships and contributing to optimized therapy.

## 5. Conclusions

To date, it is unclear whether COVID-19 represents an actual risk factor for the development of SSNHL. Nonetheless, according to the present systematic review and meta-analysis, about one third of SSNHL patients were positive for SARS-CoV-2 infection at the time of presentation in 2020–2021. Moreover, as a preliminary report of literature data regarding possible differences in epidemiological features of SSNHL during the pandemic, a trend toward younger onset age seems to have characterized SSNHL during the COVID-19 outbreak. This finding, although just in the form of a statistical trend, may outline a different pathophysiological setting during the pandemic period, in which the infectious etiology may partially explain the younger cases.

In the considered studies, the treatment modality was in line with current international guidelines. The outcomes in the study period, with audiological improvement achieved for more than half of the subjects, did not differ from those observed before the pandemic outbreak.

This review highlights the need for more methodologically robust data regarding SSNHL during the COVID-19 pandemic. Further prospective analyses on larger series and a long-term follow-up on COVID-19-related SSNHL cases are necessary to address the open questions regarding: (i) the causative link between COVID-19 infection and SSNHL and (ii) the long-term outcome of COVID-19-related hearing loss cases.

## Figures and Tables

**Table 1:** Studies included in the review and their quality according to the National Institutes of Health Quality Assessment Tool for Observational Cohorts and Cross-Sectional Studies [19].

Author; Year	Study Type	Country	PYP Pre-PYP (Month/Year)	Purpose	Results	PYP Visits	PYP N SSNHL Cases (Incidence)	Pre-PYP N Visits	Pre-PYP N SSNHL (Incidence)	Quality [19]
Parrino et al. [17]; 2022	ORS	Italy	03/20–02/21 03/19–02/20	To assess the impact of COVID-19 on SSNHL and vestibular disorders.	SSNHL during the pandemic seemed worse in terms of PTA with a higher incidence of associated vestibular involvement.	2761	34 (1.23%)	3446	27 (0.78%)	Good
Fidan et al. [23]; 2021	ORS	Turkey	04/20–09/20 04/19–09/19	To measure the incidence of SSNHL presenting at a clinic during the pandemic and pre-pandemic period.	Increased incidence of SSNHL during the COVID-19 widespread compared to the same interval of the prior year.	NR	68 (8.5/100’000)	NR	41 (5.2/100’000)	Fair
Aslan et al. [27]; 2021	OPS	Turkey	04/20–04/21 01/19–01/20	To evaluate the relationship of SSNHL and Bell’s palsy in COVID-19 patients.	No relationship between COVID-19 and cases of SSNHL and Bell’s palsy was observed.	NR	42	NR	49	Fair
Tsuda et al. [24]; 2021	ORCCS	Japan	04/20–03/20 NR	To compare the efficacy of ITS and IVS therapy as initial treatment for SSNHL during the COVID-19 pandemic.	ITS administration can be considered as the first line of treatment for SSNHL in the context of widespread COVID-19.	NR	68	NR	NR	Fair
Yaseen et al. [25]; 2021	ORS	Iraq	12/20–06/21 NR	To assess the demographic, clinical, and treatment outcomes of SSNHL in COVID-19 subjects.	Majority of COVID-19-related SSNHL cases presented within 1 week of onset, with bilateral outnumbering unilateral cases. Tinnitus was the most common associated symptom. Steroid treatment achieved improvement in 50% of the cases.	NR	26	NR	NR	Fair
Swain et al. [28]; 2021	OPS	India	03/20–08/20 NR	To investigate the incidence of SSNHL in COVID-19 patients.	Patients with COVID-19 infections have a chance of hearing loss.	NR	16	NR	NR	Poor
Jin et al. [26]; 2021	ORS	China	02/20–04/20 2017–2020	To evaluate the impact of COVID-19 on ENT diseases.	COVID-19 may cause tinnitus or sudden deafness for people with or without vascular disease.	NR	73	NR	140	Fair

Abbreviations: COVID: coronavirus infectious disease; ENT: ear, nose, and throat; PYP: pandemic year period; SSNHL: sudden sensorineural hearing loss; N: number; NR: not reported; ORS: observational retrospective study; OPS: observational prospective study; ORCCS: observational retrospective case-control study; ITS: intratympanic steroids; IVS: intravenous steroids.

**Table 2:** PYP demographic and clinical information, PTA, administered treatment, and SARS-CoV-2 swab results.

Author; Year	Mean Age ± Standard Deviation at Onset (Years)	Sex (M/F, No. Cases)	Associated Symptoms (No. Cases)	Mean PTA (dB) ± Standard Deviation	PYP Treatment (No. Cases)	Mean PTA (dB) ± Standard Deviation after Treatment	Recovery/No Recovery (No. Cases)	MRI Findings (No. Cases)	No. Cases SARS-CoV-2 +/Total
Parrino et al. [17]; 2022	56.2 ± 18.5	22/20	D (15)	61.2 ± 24.4	OS (27); IVS (10); OS + ITS (1)	50.4 ± 25.6	22/12	NR	2/5
Fidan et al. [23]; 2021	51.7 ± 18.6	37/31	NR	NR	OS	NR	NR	NR	39/68
Aslan et al. [27]; 2021	45.95 ± 15.61	29/13	NR	NR	NR	NR	22/20	NR	0/42
Tsuda et al. [24]; 2021	62.18 ± 15.06	34/34	D (11)	68.77 ± 23.32	IVS (46); ITS (22); HOT (salvage therapy) (7)	NR	NR	NR	NR
Yaseen et al. [25]; 2021	39.23 ± 11.88	6/20	D (11); T (25)	50.91 ± 11.78	OS; OS + ITS	40.24 ± 15.69	21/4	No abnormalities	26/26 *
Swain et al. [28]; 2021	48.42 ± NR	11/5	D (3); T (5)	NR	OS	NR	9/7	Cochlear enhancement (10/16)	16/16 *
Jin et al. [26]; 2021	58 ± 14.18	42/31	NR	NR	NR	NR	NR	NR	NR

**Abbreviations**: D: dizziness; dB: decibel; F: female; HOT: hyperbaric oxygen therapy; ITS: intratympanic steroids; IVS: intravenous steroids; M: male; MRI: magnetic resonance imaging; NR: not reported; OS: oral steroid; PYP: pandemic year period; PPYP: pre-pandemic year period; PTA: pure tone average; T: tinnitus. Legend: * the authors only included SARS-CoV-2-positive cases in the study group.

**Table 3:** SSNHL patient’s demographic information, PTA, and administered treatment for studies that considered controls in pre-PYP.

Author; Year	Mean Age ± Standard Deviation at Onset (Years)	Sex (M/F, No. Cases)	Associated Symptoms (No. Cases)	Mean PTA (dB) ± Standard Deviation	Treatment (No. Cases)	Mean PTA (dB) ± Standard Deviation after Treatment	Recovery/No Recovery (No. Cases)
Parrino et al. [17]; 2022	55.8 ± 14.2	29/16	10 V (10)	51.9 ± 28.4	IVS (18); OS (21); OS + ITS (3)	43.1 ± 26.4	19/8
Fidan et al. [23]; 2021	67.2 ± 16.9	22/19	NR	NR	OS	NR	NR
Aslan et al. [27]; 2021	50.73 ± 17.41	32/17	NR	NR	NR	NR	25/24
Jin et al. [26]; 2021	58.75 ± 14.53	73/67	NR	NR	NR	NR	NR

**Abbreviations**: dB: decibel; F: female; ITS: intratympanic steroids; IVS: intravenous steroids; M: male; NR: not reported; OS: oral steroids; Pre-PYP: pre-pandemic year period; PTA: pure tone average; V: vertigo.

## Data Availability

The data presented in this study are available on request from the corresponding author.
